# Environmental novelty exacerbates stress hormones and Aβ pathology in an Alzheimer’s model

**DOI:** 10.1038/s41598-017-03016-0

**Published:** 2017-06-05

**Authors:** Kimberley E. Stuart, Anna E. King, Carmen M. Fernandez-Martos, Mathew J. Summers, James C. Vickers

**Affiliations:** 1 Wicking Dementia Research and Education Centre, Faculty of Health, University of Tasmania, Tasmania, 7000 Australia; 2 School of Social Sciences, University of the Sunshine Coast, Sippy Downs, Queensland, 4556 Australia

## Abstract

Cognitive stimulation has been proposed as a non-pharmacological intervention to be used in primary, secondary and tertiary prevention approaches for Alzheimer’s disease. A common familial Alzheimer’s disease transgenic model showed heightened levels of the stress hormone, corticosterone. When exposed to periodic enhanced cognitive stimulation, these animals demonstrated further heightened levels of corticosterone as well as increased Aβ pathology. Hence, Alzheimer’s disease may be associated with hypothalamic-pituitary-adrenal (HPA) axis dysfunction, causing stimulatory environments to become stress-inducing, leading to a glucocorticoid-pathology cycle contributing to further Aβ release and plaque formation. This finding suggests that stimulation-based interventions and local environments for people with Alzheimer’s disease need to be designed to minimise a stress response that may exacerbate brain pathology.

## Introduction

The ageing of the global population is accompanied by an increasing substantial social and financial impact of ageing-related diseases that cause dementia. Among elderly populations, Alzheimer’s disease (AD) is the most common cause of dementia^1^. Research has consistently demonstrated that the environment influences the brain both structurally and functionally over the lifetime^2^. In this respect, among modifiable factors that are associated with a reduced risk of developing dementia, is living a cognitively engaged life. Indeed, those who engage in high levels of cognitive activity have approximately half the risk of developing dementia than those who engage in low levels^3^. The cognitive reserve theory (CR) posits that a high-level of intellectual engagement over the life promotes strong and efficient neuronal connections that enables the neural system capacity to withstand a greater degree of pathological insult before clinical expression of the disease process emerges^4^.

In animal models, the building of cognitive reserve can be modelled experimentally through an environmental enrichment (EE) paradigm. EE describes the manipulation of an animals’ environment in order to generate novelty and complexity, and heighten cognitive, sensory, and physical stimulation^5^. EE applied in early-life has been found to boost cognitive performance in familial AD (FAD) transgenic mouse models to the level of healthy wildtypes (Wt). However, EE is reported to have a variable effect on Aβ pathological burden in transgenic mice expressing human FAD-related gene mutations, with reported decrease^6–8^, no change^9–11^, or increase^12, 13^ in Aβ load. Alternatively, it is possible that more complex, and novel stimulation may be required to buffer AD-related neuropathology^3^. Further, individuals are more likely to take up interventions to delay dementia onset in mid- to late-life, or even after the onset of early symptoms of dementia. In a previous investigation, we demonstrated some beneficial effects of mid-life EE on cognitive function, with no changes to Aβ neuropathological burden in FAD model mice^14^. Therefore, we sought to examine whether added periodic augmentation of more novel stimulation (EE+) in FAD animals from mid-life (6 months of age), would produce findings consistent with previous reports of reduced Aβ burden following EE^6–8^. In order to examine EE in mid to later-life, mice were raised in standard housing (minimal stimulation; SH) conditions until 6 months of age, and then randomly assigned to SH, environmental enrichment (EE) or enhanced environmental enrichment (EE+) conditions, where the animals remained until 12 months of age. Mice in EE lived in a larger cage to that of SH, and enrichment objects were present in the cage (wooden and plastic blocks of differing shapes and sizes, platforms, a ball, running wheel, and a mouse hut). Mice in the EE+ condition lived in EE, but were also exposed to a novel, larger cage three times per week that contained novel enrichment objects (e.g. steps, ladders, large running wheel, mazes, toys of different textures; changed weekly).

## Results

### Mid-life EE+ was associated with increased Aβ plaque pathology in the hippocampus

**Figure 1:**
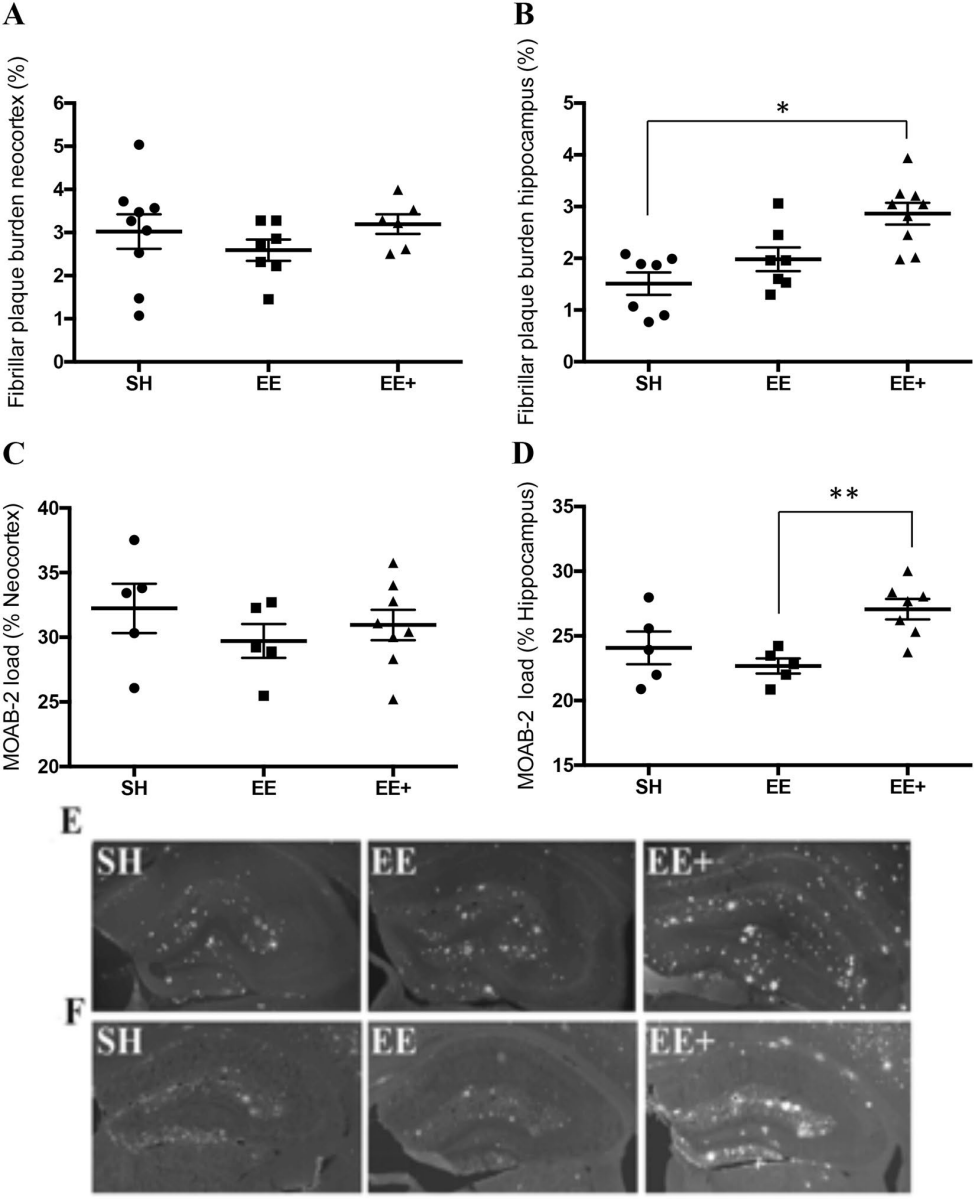
Aβ plaque load in neocortex and hippocampus in APP/PS1 mice exposed to differential housing. (**A**) Thioflavin-S staining of fibrillar and dense core Aβ plaques demonstrated no significant difference in plaque load in neocortex between the housing groups, F_(2, 19)_ = 0.78, *p* = 0.47. (**B**) A significant effect of housing was detected on Thioflavin-s plaque load in the hippocampus, F_(2, 19)_ = 4.80, *p* = 0.02. Bonferroni adjusted *post hoc* tests revealed APP/PS1 mice housed in EE+ had higher hippocampal Aβ fibrillar plaque burden when compared to those in SH (*p* = 0.02). (**C**) MOAB-2 immunolabelling demonstrated no significant differences across housing groups in Aβ_1-42_ load, F_(2, 18)_ = 0.65, *p* = 0.54. (**D**) A significant effect of housing condition was detected on hippocampal MOAB-2 load, F_(2, 18)_ = 7.78, *p* = 0.005. Bonferroni adjusted *post hoc* tests showed EE+ mice to have a significantly higher hippocampal MOAB-2 load relative to mice in SH. SH (*n* = 9), EE (*n* = 7), EE+ (*n* = 6) *p < 0.05, **p < 0.01, ***p < 0.001. (**E**) Representative 10x Thioflavin-s staining in hippocampus of APP/PS1 mice for each housing group. (**F**) Representative 10x MOAB-2 staining in hippocampus of APP/PS1 mice for each housing group.

Following the 6-month intervention, when mice were 12 months of age, we hypothesised exposure to the EE+ intervention would potentially be associated with a reduction in Aβ plaque burden. We analysed fibrillar plaque load by Thioflavin-s (Fig. 1E) staining and by immunostaining of human unagreggated, oligomeric, and fibrillar forms of Aβ_1-42_ with the MOAB-2 antibody (Fig. 1F), in the neocortex and hippocampus of APP/PS1 mice. No significant alterations to Aβ load in the neocortex as a function of housing condition was detected for either Thioflavin-s or MOAB-2 labelled Aβ plaques (Fig. 1A,C). In contrast, there was a large (*d* = 1.08) and significant increase in hippocampal fibrillar Aβ plaque load in APP/PS1 mice exposed to the EE+ condition (Fig. 1B), and this effect was also detected for Aβ_1-42_ immunolabelling (Fig. 1D; *d* = 1.83).

### Mid-life EE+ was associated with increased *Aβ*_*1-42*_ in neocortex

**Figure 2:**
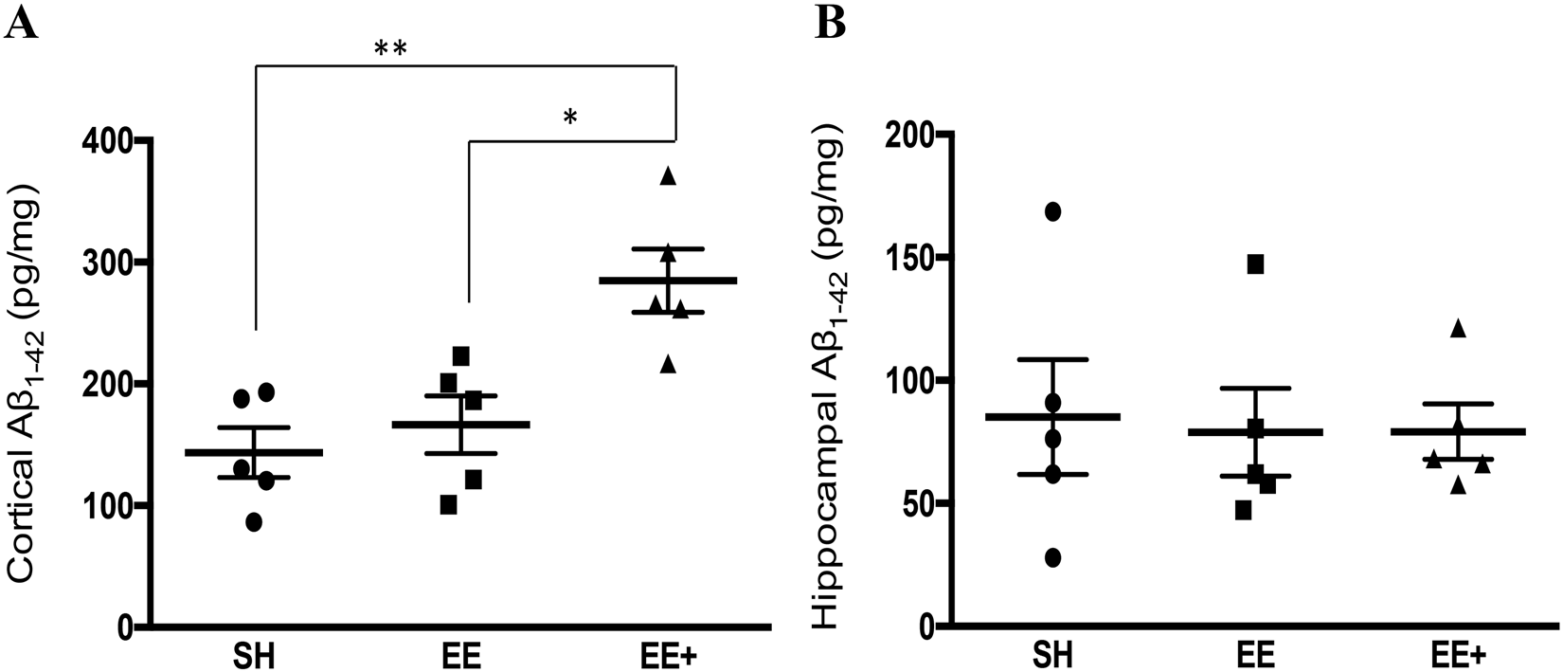
*Aβ*_*1-42*_ levels in neocortex and hippocampus in APP/PS1 mice across differential housing conditions. (**A**) Overall, there was a significant effect of housing condition on cortical *Aβ*_*1-42*_ levels, F_(2, 12)_ = 10.41, *p* = 0.002. Follow-up *post-hoc* tests were performed with Bonferroni correction applied. The EE+ group had significantly higher cortical *Aβ*_*1-42*_ compared with both SH (*p* = 0.003) and EE (*p* = 0.012). (**B**) No significant differences were detected in hippocampal *Aβ*_*1-42*_ levels between housing conditions, F_(2, 12)_ = 0.04, *p* = 0.96. SH (*n* = 5), EE (*n* = 5), EE+ (*n* = 5) *p < 0.05, **p < 0.01, ***p < 0.001.

Next, we examined levels of soluble *Aβ*_*1-42*_ in the neocortex and hippocampus, and found an overall significant effect of housing condition on *Aβ*_*1-42*_. APP/PS1 mice housed in EE+ showed a large (*d* = 1.89) and significant increase in *Aβ*_*1-42*_ in neocortex, compared to mice from SH or EE (Fig. 2A). However, in contrast to the finding of an increase in Aβ plaque deposition in the hippocampus, this effect was not observed for *Aβ*_*1-42*_ levels in the hippocampus (Fig. 2B). These data suggest that EE+ is associated with increases in Aβ levels in the neocortex, and increased deposition of Aβ in the hippocampus.

### APP/PS1 mice demonstrated elevated levels of corticosterone

**Figure 3:**
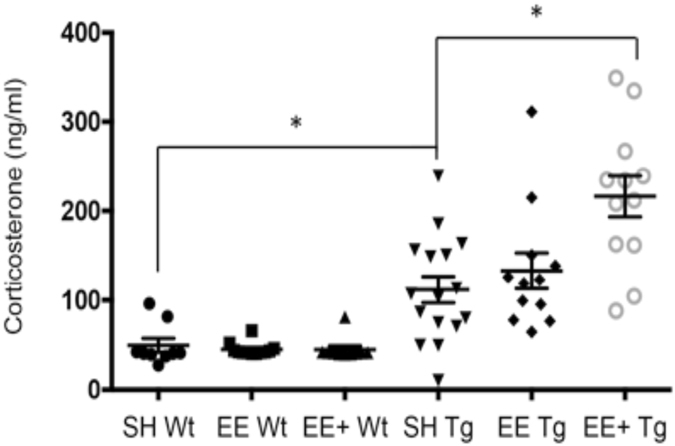
Differential corticosterone levels according to genotype and housing condition. APP/PS1 mice in SH had significantly higher levels of corticosterone in blood serum compared to healthy Wt control mice at 12 months, *t*_(24)_ = 2.34, *p* = 0.03. There was a significant interaction effect of genotype × housing on levels of corticosterone, F_(2, 66)_ = 3.76, *p* = 0.03. In Wt mice, there was no significant effect of housing on corticosterone, F_(2, 28)_ = 0.31, *p* = 0.74. Housing condition produced a significant effect on corticosterone levels in APP/PS1 mice, F_(2, 38)_ = 4.8, *p* = 0.01. Bonferroni adjusted *post hoc* tests revealed EE+ mice had significantly higher corticosterone levels compared to SH (*p* = 0.02). Moreover, EE+ mice had higher corticosterone levels than mice housed in EE (*p* = 0.049), however, this was no longer significant when Bonferroni correction was applied (*p* = 0.057). SH Wt (*n* = 9), EE Wt (*n* = 12), EE+ Wt (*n* = 10); SH Tg (*n* = 17), EE Tg (*n* = 12), EE+ Tg (*n* = 12) *p < 0.05, **p < 0.01, ***p < 0.001.

While our assumption had been that placing animals in an enhanced EE condition may have augmented cognitive and physical stimulation, it was also possible that this exposure had broader physiological effects that may have led to increased Aβ pathology, and that the FAD background also contributed to an unexpected reaction to the EE+ condition. Previous clinical studies have reported that people living with AD generally have higher blood-levels of the stress hormone, cortisol, when compared to healthy people^15, 16^. However, whether the disease process itself alters normal stress physiology, or whether stress increases vulnerability to developing AD, is unknown. It is widely accepted that stress induces activation of the hypothalamic-pituitary-adrenocortical (HPA) axis, which leads to a subsequent release of cortisol in humans, or corticosterone in rodents, from the adrenal cortex^17, 18^. Several studies, using experimental paradigms designed to induce stress, have demonstrated an accelerated onset, or faster rate of progression of AD, in FAD mouse models^19–23^. Such evidence led us to hypothesise that the novelty and complexity conferred by the EE+ condition, elevated stress levels, which may in turn have increased Aβ pathological burden in APP/PS1 mice in EE+ housing. We first compared levels of the stress hormone, corticosterone, between healthy control Wt and APP/PS1 mice from the SH condition, in order to determine whether the presence of the APP/PS1 transgene was associated with elevated corticosterone. Corticosterone levels in serum were significantly increased in 12-month old APP/PS1 mice compared to healthy Wt mice, which represented a large effect (*d* = 1.09) (Fig. 3). This finding supports the hypothesis of HPA axis dysfunction in AD.

### Mid-life EE+ was associated with elevated corticosterone levels in APP/PS1 mice

Following the finding of elevated levels of corticosterone in APP/PS1 mice compared to Wt mice from SH, we included EE and EE+ conditions into the analysis. First, we found an overall significant interaction effect of genotype x housing on corticosterone levels. Due to the APP/PS1 mice demonstrating increased corticosterone levels, we analysed genotypes separately to investigate housing effects. In Wt mice, there was no significant housing condition effect on corticosterone levels. However, there was a significant and large effect of housing condition for APP/PS1 mice. While the APP/PS1 mice from SH exhibited increased corticosterone levels relative to Wts, APP/PS1 mice from EE+ showed further elevated corticosterone levels, being significantly higher compared to APP/PS1 mice from both the SH and EE conditions (*d* = 1.05) (Fig. 3).

## Discussion

Our study has shown that periodic augmentation of EE conditions in APP/PS1 mice may have a deleterious effect on Aβ pathology, and this was associated with heightened levels of corticosterone, in a FAD model that already showed elevated stress hormone levels over Wt animals of a similar age. In contrast, sustained exposure to regular EE conditions did not affect corticosterone levels or amyloid pathology, indicating a potential capacity to habituate to long-term changes in the housing environment. While APP/PS1 animals housed in EE+ had increased Aβ pathology along with elevated corticosterone, no significant behavioural differences were detected on the Y maze test of spatial short-term memory, or the Barnes maze test of spatial learning and long-term memory relative to APP/PS1 mice housed in SH or EE (data not shown). The finding of increased pathological burden without cognitive dysfunction may offer support for the cognitive reserve theory^4^, however following up at later time points when neural resources are reduced, would be of interest.

Clinical studies have included reports of people living with AD to generally have higher levels of blood cortisol when compared to healthy people^15, 16^. Moreover, elevated levels of corticosterone has been reported previously in a FAD mouse model^24^, and Aβ accumulation has been associated with increased sensitivity of the HPA axis^23^. The present study confirms HPA axis dysfunction in an animal model of early AD. Several studies have demonstrated using experimental paradigms designed to induce acute stress (e.g. by restraint, loud noise, and bright light) an accelerated onset, or faster rate of progression of AD, in FAD mouse models^19–23^. However, in the current report, we found an association between the periodic stimulation condition and increased corticosterone. APP/PS1 mice display Aβ pathology from 4-5 months of age^25^, in the hippocampus and neocortex, and associated damage to synapses^26^, and the hippocampus directly regulates the HPA axis^27–29^. Green and colleagues^30^ speculated that, in AD, glucocorticoid feedback to the hippocampus may be lost, leading to an increased stress response. The increasing levels of glucocorticoid may further accelerate pathological processes, acting as a cycle of disease process progression. Notably, Wt mice did not show increased corticosterone levels after exposure to EE+, and, thus, were not stressed by the periodic stimulation of the EE+ condition. In contrast, FAD gene-linked deposition of Aβ in the hippocampus and synaptic damage in transgenic animals may lead to initial dysregulation of the HPA-axis, which contributed to a heightened stress response following periodic stimulation in the EE+ condition. Whereby, this periodic stimulation in EE+ may stimulate a harmful cascade, with subsequent increases in Aβ leading into an increased stress response to EE+, further increasing circulating corticosterone, and further Aβ deposition within the hippocampus.

The finding that elderly who enter a nursing home demonstrate more rapid cognitive decline compared to those who remain in the community^31^, together with the findings of this study, raise questions into the effect of novel environments for people with AD on disease progression. Moreover, the findings presented here bring into question cognitive interventions for the elderly, and for people living with AD in particular. An intervention aimed at delaying the progression of the clinical syndrome, dementia, is urgently needed given the rapidly increasing ageing population. As there are no current efficacious pharmacological treatments available, non-pharmacological interventions such as cognitive stimulation, training, and rehabilitation are of current interest. Such interventions have demonstrated various levels of efficacy, although modest sized effects are typically reported^32^. To our knowledge, the effects of cognitive intervention on stress in people living with AD has not been examined. Moving forward, this will be an important point of investigation, as cognitive interventions for people living with AD may have limited beneficial effect, and may potentially induce stress leading to an increased progression of disease processes. Likewise, given that Aβ pathology may be present in the brain many years before overt dementia symptomology^33^, it will be important to examine the role that stress may have in facilitating further pathology, and that primary and secondary prevention strategies that involve cognitive stimulation are designed to minimise elevated stress.

## Methods

### Animals and EE protocol

In the current study, we used a transgenic mouse model of AD that most closely resembles early-stage AD^26^. Male mice expressing chimeric mouse/human amyloid precursor protein (APP) and mutant human presenilin 1(PS1) on C57BL/6 background [B6.Cg-Tg (APPswe, PSEN1_dE9_) 85Dbo/J] (APP/PS1; Jankowsky *et al*.^25^ and littermate wildtype (Wt) control mice were housed in standard housing (SH) conditions (4-5 mice per 30 × 30 × 14 cm cage, *ad libitum* access to food and water, an igloo, one small wooden stick and one tissue) until 6 months of age. At 6 months, mice were randomly assigned to SH, EE, or EE+ conditions for the following 6 months. Mice assigned to the EE and EE+ housing conditions lived in a larger 60 × 30 × 14 cm cage with the contents of the SH cage and the addition of enrichment objects (wooden and plastic blocks of differing shapes and sizes, platforms, a ball, running wheel, and a mouse hut). The EE+ condition involved the mice being exposed to a larger (36 × 49 × 22 cm) cage three times per week that contained novel enrichment objects (changed weekly). Housing conditions were maintained until the 12-month end-point. All experimental procedures presented here were performed in accordance with the Australian Code of Practice for the Care and Use of Animals for Scientific Purposes, and approved by the University of Tasmania Animal Ethics Committee (A13253).

### Tissue collection

Mice were terminally anaesthetized first with gas anaesthesia (isoflurane) followed by sodium pentobarbitone (100 mg/kg delivered intraperitoneally). For histological analysis, animals were perfused transcardially with 4% paraformaldehyde (PFA) in 0.1 M phosphate buffered saline (PBS pH 7.4). Post-mortem brains were transferred to 18% then 30% sucrose solutions overnight. Brains were cut on a cryostat (Leica CM 1850) in 40 μm coronal sections. APP/PS1 mice used for the quantification of human soluble *Aβ*_*42*_, were terminally anaesthetized as above, and perfused transcardially with PBS (0.1 M). Post-mortem brains were removed and the cortex and hippocampus were dissected and immediately frozen in liquid nitrogen. Cortex and hippocampal samples were stored at −80 °C for later analysis.

### Aβ fibrillar plaque deposition

Thioflavin-S (Sigma-Aldrich) staining was performed in order to visualise fibrillar and dense-core Aβ plaques^34^, ten serial sections evenly spaced throughout the rostrocaudal axis of the brain from bregma 2.0–3.0 mm according to the stereotaxic mouse brain atlas^35^ were incubated in the solution (0.125% Thioflavine-S diluted in 60% absolute ethanol with 40% 0.01 M PBS) for 3 minutes at room temperature, and washed for 2 × 1 minute in 50% absolute ethanol and 50% 0.01 M PBS solution, followed by 3 × 10 minute washes in 0.01 M PBS. MOAB-2 immunostaining was performed in order to visualize human fibrillar, as well as unaggregated and oligomeric forms of Aβ_42_^14, 36^. Ten serial sections evenly spaced throughout the rostrocaudal axis of the brain from bregma 2.0–3.0 mm were immunolabelled with the MOAB-2 antibody (1:2000; Novus Biologicals), and the labelling was visualized by inclubation in Alexa-fluorophore conjugated secondary antibody (1:1000; Molecular Probes, goat anti-mouse IgG2b-546). Sections were mounted using Dako fluorescent mounting medium.

### Image acquisition

In order to determine Aβ plaque load in the neocortex and hippocampus, images were taken on a Leica DM fluorescence microscope on a 10x objective with NIS Elements imaging software. The left side of the cortex was imaged from the midline to the rhinal fissure from bregma 2.0–3.0 mm of 10 sections per animal. Images of the whole hippocampal formation were taken between bregma position −1.22 and −2.46 mm of 3–5 sections per animal. Aβ plaque load (percentage area Thioflavin-S positive) was calculated by random forest segmentation for one hemisphere of the neocortex and the whole hippocampal formation. Whereby, a custom plugin for ImageJ was applied to the images, which automatically segmented images as plaques or background pixels by random forest classification^37^. The percent area stained by Thioflavin-S in the neocortex and hippocampus was calculated by dividing the labelled area by the total area analysed.

### Aβ_42_ ELISA

For the quantitation of human *Aβ*_*1-42*_, a sandwich antibody ELISA was performed according to the manufacturer’s instructions (KHB3441, Invitrogen). The right side of the neocortex and hippocampus were homogenized in RIPA buffer (Sigma) containing a protease (Roche diagnostics) and phosphatase inhibitor cocktail (AG Scientific). The samples were centrifuged for 15 minutes at 13000 RPM, rotated for a further 30 minutes, and centrifuged again at 4 °C for 15 minutes at 13000 RPM. The resulting supernatant was removed and stored at −80 °C for protein analysis. The protein concentrations of samples were determined using the Bradford assay. Duplicate neocortex and hippocampus samples from APP/PS1 mice (*n* = 5/group) were diluted in the buffer provided (1:50). Soluble human *Aβ*_*1-42*_ levels were normalized to total protein levels and expressed as picogram of *Aβ*_*1-42*_ content per milligram of total protein (pg/mg). Optical densities were read at 450 nm on a microplate reader (SpectraMax, Molecular Devices), and concentrations of *Aβ*_*1-42*_ were determined by comparison to the standard curve using a 4-parameter algorithm.

### Corticosterone ELISA

In order to determine if there were differences in levels of stress between the housing conditions and genotypes, blood was collected at time of perfusion by cardiac puncture, performed at the start of the dark period (3 p.m.). Duplicate serum samples were diluted at 1:100 in the buffer provided, and levels of serum corticosterone were measured by a commercial ELISA kit (ab108821; Abcam) according to manufacturer instructions. Optical densities were read at 450 nm on a microplate reader (SpectraMax, Molecular Devices), and concentrations of corticosterone were determined by comparison to the standard curve using a 4-parameter algorithm.

### Statistical analysis

Statistical analyses were performed using independent t-test and two-way ANOVA using IBM SPSS (Version 20). A statistically significant two-way ANOVA was followed up by *post hoc* tests. Variables considered were genotype (Wt or Tg) and housing condition (SH, EE, or EE+). Values of *p* < 0.05 for differences between group means were classified as statistically significant. The magnitude of differences between the means were reported as Cohen’s *d*.

### Data availability

The datasets generated during and/or analysed during the current study are available from the corresponding author on reasonable request.
